# Integration of Novel Low-Cost Colorimetric, Laser Photometric, and Visual Fluorescent Techniques for Rapid Identification of Falsified Medicines in Resource-Poor Areas: Application to Artemether–Lumefantrine

**DOI:** 10.4269/ajtmh.14-0832

**Published:** 2015-06-03

**Authors:** Michael D. Green, Dana M. Hostetler, Henry Nettey, Isabel Swamidoss, Nicola Ranieri, Paul N. Newton

**Affiliations:** Division of Parasitic Diseases and Malaria, Center for Disease Control, Atlanta, Georgia; Department of Pharmaceutics and Microbiology, School of Pharmacy, College of Health Sciences, University of Ghana, Legon, Ghana; Forensic Chemistry Center, U.S. Food and Drug Administration, Cincinnati, Ohio; Lao-Oxford-Mahosot Hospital-Wellcome Trust Research Unit, Microbiology Laboratory, Mahosot Hospital, Vientiane, Lao PDR; Centre for Tropical Medicine and Global Health, Nuffield Department of Medicine, University of Oxford, Churchill Hospital, Oxford, United Kingdom

## Abstract

The availability of falsified antimalarial drugs can be reduced with effective drug regulatory agencies and proper enforcement. Fundamental to these agencies taking action, rapid identification must be made as soon as they appear in the market place. Since falsified antimalarials occur mostly in developing countries, performing drug analysis presents itself with unique challenges. A fundamental factor in choosing a useful technique is affordability and simplicity. Therefore, we suggest a three-tiered drug evaluation strategy for identifying a falsified drug in resource-poor areas. Tier I is a simple comparison of a tablet's weight and dimensions with official specifications. Tier II uses inexpensive photometric devices (laser and fluorescence) to evaluate a tablet. Suspicious samples from Tier I and II assessments are then subjected to a colorimetric assay for active ingredients identification and quantification. In this article, we evaluate a novel colorimetric assay for the simultaneous assessment of both lumefantrine and artemether in co-formulated Coartem^™^ tablets, and integrate the method with two novel, low-cost, fluorescence and laser photometric devices. Image analysis software is used for the assessments. Although artemether–lumefantrine is used as an example, the strategy may be adapted to other medicines.

## Introduction

Low-income countries bear the highest proportion of deaths caused by human immunodeficiency virus/acquired immune deficiency syndrome (HIV/AIDS), tuberculosis, and malaria. In 2012, an estimated 627,000 deaths were attributed to malaria; with the disease occurring mostly in African children.^1^ Malaria can be cured with effective antimalarial drugs but due to the growth of *Plasmodium falciparum* resistance, traditional antimalarials such as chloroquine and sulfadoxine/pyrimethamine are no longer recommended. Although the artemisinins are the most rapidly acting of all antimalarial drugs,^2^ potential resistance has led to the abandonment of oral artemisinin monotherapy. Therefore, this class of antimalarial is combined with a partner drug, such as lumefantrine, amodiaquine, piperaquine, or mefloquine, possessing a longer half-life. This formulation, termed artemisinin combination therapy (ACT) is recommended by the World Health Organization (WHO) as first-line treatment in most malaria-endemic countries.^3^ An ACT being very widely purchased and distributed by international and national organizations globally combines lumefantrine (long half-life drug) with artemether (artemisinin derivative) into a fixed-dose tablet (AMLF). The first fixed-dose ACT, meeting the WHO prequalification criteria for efficacy, safety, and quality is the brand Coartem^™^, an AMLF drug manufactured by Novartis (Basel, Switzerland).^4^ As a result of the expanding market in Africa, falsified (counterfeit) AMLF (Coartem) has already been reported in many west and central African countries.^5^,^6^

Low-income countries with high malaria burden are easy targets for falsified (term used to distinguish from the term counterfeit medicines that invokes intellectual property issues) drug manufacturers. Although these countries are relatively poor, the prevalence of the disease contributes to a high volume of sales, thereby making these areas lucrative locations for the counterfeit trade. Also, weak drug regulatory and enforcement agencies enable the counterfeiters to proliferate. The scarcity and high cost of some medicines and unregulated markets are also major contributors to the problem. It is imperative that a falsified medicine be identified as quickly as possible so that regulatory agencies and enforcement officials can take immediate action, assuming that drug quality is a priority issue and these agencies are adequately funded to take the necessary action. Standard methods for drug quality evaluation include high-performance liquid chromatography (HPLC) and spectroscopy. Because of the high cost of instruments, expensive maintenance, lack of expertise, and less-than-ideal operational conditions, utilization of these techniques are not practical in low-income countries. Spectroscopic devices such as Raman and near-infrared (NIR) can quickly and accurately scan a sample and determine its legitimacy.^7^–^10^ These techniques do not destroy the sample and are fairly easy to operate. The main disadvantages of NIR and Raman spectroscopy are that the NIR spectra are often complicated by broad and overlapping absorption bands resulting from molecular overtones and combination vibrations, while Raman spectra may be complicated by the presence of interfering fluorescent compounds. As a result, specialized software may be required to interpret or detect subtle spectral differences. Although not as expensive as HPLC, the spectroscopic devices are still outside the realm of affordability of many low-income countries and have not been fully and independently evaluated. There are a myriad of various field methods that are easily adaptable in low-income countries, depending on their infrastructure and resources. The Institute of Medicine has recently published a report on “Countering the Problem of Falsified and Substandard Drugs” that contains a very comprehensive review of drug detection techniques^11^ and several reviews on simple counterfeit drug field-detection techniques.^12^–^14^

Portable field kits utilizing colorimetric techniques have long been the choice of investigators for quickly identifying poor quality medicines. Colorimetry has also been effectively used to aid in identifying a falsified drug based on the absence of the active ingredient. When counterfeit artesunate tablets began to appear in southeast (SE) Asia,^15^–^17^ colorimetric assays were developed to rapidly assess drug authenticity.^18^–^21^ Early methods involved a reaction of Fast Red TR salt (FRTR) with the artemisinins (artesunate, dihydroartemisinin, and artemether) to produce a specific yellow color.^18^,^19^ The Global Pharma Health Fund (GPHF) incorporated the FRTR colorimetric method for artesunate and artemether into the GPHF-Minilab^®^; a field-adapted kit containing all required supplies and reagents for colorimetric and thin-layer chromatographic analysis of essential drugs used in developing countries.^22^ The FRTR technique was successfully used in Laos and Ghana to evaluate artesunate quality,^13^,^23^ but as the new ACTs were becoming recommended treatment, oral artemisinin monotherapy was phased out. Many of the new ACTs combined the artemisinins with yellow-colored partner drug such as lumefantrine and amodiaquine in a fixed-dose co-formulation, thereby making the FRTR test obsolete. As an alternative, Ioset and Kaur produced a colorimetric test that resulted in a blue or pink product when positive for the artemisinins.^21^ One of the most widely used ACTs is the artemether–lumefantrine co-formulated tablet. Nyarko and Nettey have combined a colorimetric test with image analysis software to quantitatively assess lumefantrine and artemether in tablets collected in Ghana.^24^ Although colorimetry as well as thin-layer chromatography (TLC) are relatively inexpensive, spectroscopic (Raman, NIR) methods have useful advantages in resource-challenged countries. These advantages include a high throughput, high accuracy, and no sample preparation that eliminates the use of solvents, chemicals, or labware. These methods are usually qualitative, mostly for rapid screening of suspicious medicines. Incorporating multivariate analysis techniques with NIR and Raman spectral comparisons, falsified artesunate tablets have been successfully identified with 100% accuracy.^8^,^10^ Substituting complex multivariate analysis with a simple spectral correlation value (1.00 = a perfect spectral match), our laboratory conducted a comparison of NIR and Raman analysis on 24 confirmed falsified Guilin Pharmaceutical Co. (Shanghai, China) brand artesunate tablets. Raman analysis demonstrated both sensitivity and specificity of 100%, while NIR analysis resulted in sensitivity and specificity of 80% and 100%, respectively. In regard to AMLF tablets, the Raman spectra showed a large distinct band correlating to the relative concentration of the lumefantrine active ingredient present in the tablet. This characteristic has the potential to be useful for quantitative analysis of lumefantrine in AMLF tablets of various brands. Because of its portability and lower cost, we suggest Raman spectroscopy to be better suited for field analysis. Table 1 lists the types of tests suggested for use in the field and are grouped by tiers; with Tier I being the least complex in terms of equipment, training, and affordability and Tiers II and III increasing in complexity. Tier I consists of simple physical measurements (e.g., tablet weight and dimensions) where many falsified medicines outside the specification limits can be identified. Tier II suggests the use of rapid scanning techniques using photometric devices followed by techniques requiring more advanced technical expertise such as colorimetric assays and TLC (Tier III). The suggested strategy is to initially assess tablets using Tier I and II techniques, which are not destructive to the sample and does not require the use of chemical reagents or solvents. Tier III techniques are reserved for suspicious tablets failing Tiers I and II assessments, thereby conserving the transport and use of toxic and/or flammable chemicals used for Tier III. Quantitative Tier III assessments are important in identifying counterfeits containing active ingredients, since substandard amounts can contribute to drug resistance. In lieu of expensive Raman or NIR devices listed in the Tier II group in Table 1, we propose the use of two novel, low-cost, handheld, and nondestructive counterfeit drug screening devices: the counterfeit detection device version 3 (CD-3)^25^ developed at the U.S. Food and Drug Administration (FDA) and a laser-emitting photometric device counterfeit drug indicator (CoDI) developed by Michael D. Green.

The CD-3 is a compact handheld device with a series of light-emitting diodes (LEDs) of various wavelengths. A sample is illuminated by a selected wavelength and the image is visualized with a charged-couple device (CCD). Depending on the formulation, the tablet may reflect a particular wavelength differently (fluorescence) resulting in characteristic intensities for a particular product. By comparing with an authentic sample, suspicious products are visually distinguishable. Samples may be visualized without compromising the blister pack containing the tablets. The CD-3 is relatively inexpensive (< 1,000 USD), robust, accurate, and easy to operate. The CD-3 was recently evaluated on counterfeit and genuine artesunate tablets collected in Laos, demonstrating 100% positive predictive value and 97.4% negative predictive value.^25^ The features of the CD-3 can also be used to distinguish counterfeit packaging because of the use of different quality inks as well as the appearance of covert markings. Since falsified medicines may include repackaged expired product, this feature of the CD-3 is extremely useful.

The CoDI is a battery-operated handheld device incorporating a 405-nm laser, a photoresistor, and digital voltmeter as the main components. A tablet is placed in the sample well, and the amount of laser light passing through the tablet is measured using the photoresistor and voltmeter. The intensity of light detected by the photoresistor results from a combination of the following parameters: 1) tablet thickness, 2) tablet density, and 3) wavelength of light emitted by the tablet matrix (fluorescence) relative to the spectral response of the cadmium sulfide (CdS) photoresistor. Use of colored filters in conjunction with the fluorescent properties of the tablet provides a unique value for the light intensity emitted through the tablet. The device is nondestructive to the tablet, low cost (< 100 USD), simple to use, and provides rapid results.

The primary objective of this article was to evaluate a three-tiered falsified drug identification strategy designed for use in resource-poor countries. A collection of falsified Coartem tablets was used for the evaluation. The sources of the tablets were not identified because of security issues. The strategy consists of an initial physical evaluation by simple measurements of tablet weights and dimensions (Tier I), followed by rapid scanning of the tablets using the CD-3 and CoDI devices (Tier II). Suspicious tablets, as determined from Tier I and II, are then subjected to a colorimetric test to identify and quantify the active ingredients (Tier III). Previously described colorimetric tests for the artemisinins required reagents not readily available in many developing countries.^18^–^21^ We have developed a simple colorimetric test for lumefantrine and artemether in a single-tablet solution using only sulfuric and acetic acid. These commonly used acids are inexpensive and stabile. Unfortunately, sulfuric acid has been frequently used in the illicit manufacture of narcotics, therefore large amounts distributed through international commerce have become controlled. Although, its availability and acquisition may limit its application in the field, the small amounts necessary for the colorimetric assay may be less problematic. Both lumefantrine and artemether are completely dissolved in acetic acid. The intensity of the inherent yellow color of lumefantrine as well as the orange/red product of an artemether/sulfuric acid reaction product is assessed using image analysis software.

The primary aim of this article was to evaluate tablet measurements (Tier I), CD-3 and CoDI (Tier II), and the colorimetric acid test (Tier III) as a rapid, simple, and low-cost strategy for identifying counterfeit Coartem tablets in resource-poor, low-income countries.

## Materials and Methods

### Physical: Weights and dimensions.

Sample tablets were weighed using an analytical balance (0.001 g precision), and tablet thickness and diameter were measured using digital calipers (0.01 mm precision). The official specification ranges for the weight and thickness of an authentic Coartem tablet are 0.228–0.252 g and 3.0–3.4 mm, respectively (Novartis, personal communication). A diameter range of 9.1–9.2 mm was determined from the measurements of 12 authentic Coartem tablets.

### Colorimetric assay: Preparation of calibration standards.

Secondary calibration standards were prepared by combining several authentic Coartem tablets and analyzing for lumefantrine (LF) and artemether (AM) content by HPLC. In brief, tablets were pulverized and a weighed portion added to a solution of 10% acetic acid in methanol. After sonication for 20 minutes, the sample was filtered through a 0.45-μm nylon membrane and injected into the HPLC. The components were separated using 150 × 4.6 mm C18, 5-μm column (Supelco Inc. Bellefonte, PA) with a mobile phase consisting of 60% acetonitrile and 40% 0.05 M sodium perchlorate (pH 2.5) at a flow rate of 1 mL/min. Detection was accomplished with ultraviolet (UV) absorbance at 210 nm. Quantitation of the sample was made by direct comparison with pure authentic LF and AM standards. Once the amount of LF and AM was established for the tablet mixture, glacial acetic acid was added to achieve a LF concentration of 36 mg/mL and an AM concentration of 6 mg/mL. These concentrations represent 120% of the active pharmaceutical ingredients (APIs) of a typical tablet (AM/LF 20 mg/120 mg) dissolved in 4 mL glacial acetic acid. Subsequent dilutions were made to obtain 100% (30 mg/mL LF, 5 mg/mL AM), 80% (24 mg/mL LF, 4 mg/mL AM), 50% (15 mg/mL LF, 2.5 mg/mL AM), 25% (7.5 mg/mL LF, 1.25 mg/mL AM), and 10% (3.0 mg/mL LF, 0.5 mg/mL AM).

### Colorimetric assay for lumefantrine and artemether calibration samples.

Of the filtered solution (0.45 μm nylon filter), 100 μL was transferred to the wells of a clear polystyrene 96-well flat-bottomed plate in triplicate. The wells were analyzed with a plate reader (SpectraMax 250; Molecular Devices, Sunnyvale, CA) set for absorbance at 420 nm for LF analysis and a digital photograph taken (PowerShot A3100 IS Camera; Canon, Lake Success, NY) after placing the plate on a light box (Visual Plus SV 450; Fujifilm, Greenwood, SC). Image analysis was performed using MVHimagePCv8 software (MVHimagePCv8, Global Systems Science, University of California). Red, green, and blue pixels for each well were measured and normalized to blue (RvsB = [Red – Blue]/[Red + Blue]) for LF and green for AM (RvsG = [Red – Green]/[Red + Green]). These image analysis values were chosen based on acceptable linearity relative to concentration. One drop (∼20 μL) of concentrated sulfuric acid was added to each well and allowed to react at room temperature for 30 minutes. The orange/red color, developed in the presence of AM, was measured by absorbance at 520 nm followed by image analysis as described above. The linearity of concentration versus absorbance and RvsB values were plotted and evaluated using Sigmaplot version 12.3 (SYSTAT Software Co., San Jose, CA). Correlations between RvsG and absorbance for LF and AM were also plotted. Specificity of the colorimetric assay was assessed by applying the procedure described above to solutions of typical doses of excipients and commonly used antimicrobials.

### Colorimetric assay procedure for tablets.

A single tablet of fixed dose LF/AM (120/20 mg) was weighed and the dimensions (diameter and thickness) were measured with a digital caliper. The tablet was placed into a glassine paper envelope and pounded to a fine powder using a pestle. The powdered sample was transferred to a glass vial and 4 mL glacial acetic acid was added. The sample was shaken vigorously for 15 seconds. After 30 minutes at room temperature, the sample was shaken again. When the undissolved particulates settled to the bottom, 100 μL filtered solution was transferred to the wells of a 96-well plate and subjected to image analysis and chemical treatment as described above for the calibration samples. The assay was applied to an authentic Coartem tablets and suspected falsified tablets.

### CD-3 analysis.

A CD-3 device was a graciously loaned to us by the FDA. The authentic Coartem tablets and suspected counterfeit tablets used for the colorimetric assay were scanned using the CD-3 prior to performing the colorimetric assay. The detection mode was set for infrared (IR) filter and scanned using the UV wavelength LED. Tablet brightness resulting from the fluorescence produced by the UV LED on the tablet surfaces were measured as “gray values” using ImageJ software (ImageJ version 1.45s; National Institutes of Health, http://imageJ.nih.gov/ij). A plot of gray values across the diameter of each tablet was determined from a digital photograph taken with the CD-3.

### CoDI laser analysis.

A prototype CoDI, as described in the introduction, was evaluated using 12 authentic and 7 counterfeited Coartem tablets as well as two other brands of AM/LF tablets from various batches. Twelve groups of five tablets of common antimalarials were also assessed using the CoDI. The samples included artesunate, chloroquine, mefloquine, and sulfadoxine/pyrimethamine of various brands or manufacturers. The authenticities of the Coartem tablets were confirmed from Raman scans and HPLC analysis. Specific operational details of the CoDI were not disclosed because of potential patent issues. Each sample tablet was placed in a compartment from which a 405-nm laser beam is emitted. The intensity of light emitted through the tablet was recorded using a photoresistor coupled to a voltmeter. A red filter was then placed over the photoresistor and the intensity rerecorded from the same tablet. A CoDI value (*W*) was calculated from the difference and ratio of light intensity (I) with and without the filter [*W* = (*I* – *I*_filter_)/(*I*_filter_/*I*)]. Box-and-whisker plots^26^ were used to compare values for each group of samples to evaluate the CoDI in terms of its specificity for Coartem. Nonparametric Mann–Whitney test (MedCalc version 13.2.0.0; MedCalc Software bvba, Osten, Belgium) was used to evaluate significant differences between the Coartem group and the other drugs analyzed.

## Results

### Physical: Weights and dimensions.

Table 2 shows the weights and dimensions of the sample tablets relative to the range expected for authentic tablets. Any tablet's measurements falling outside the acceptable range would be considered as “failed.” Except for CF #2 and CF #6, all the samples failed. This information along with the photometric results was used to determine if the sample should undergo the colorimetric test.

### Colorimetric assay.

Figure 1A

**Figure 1.**
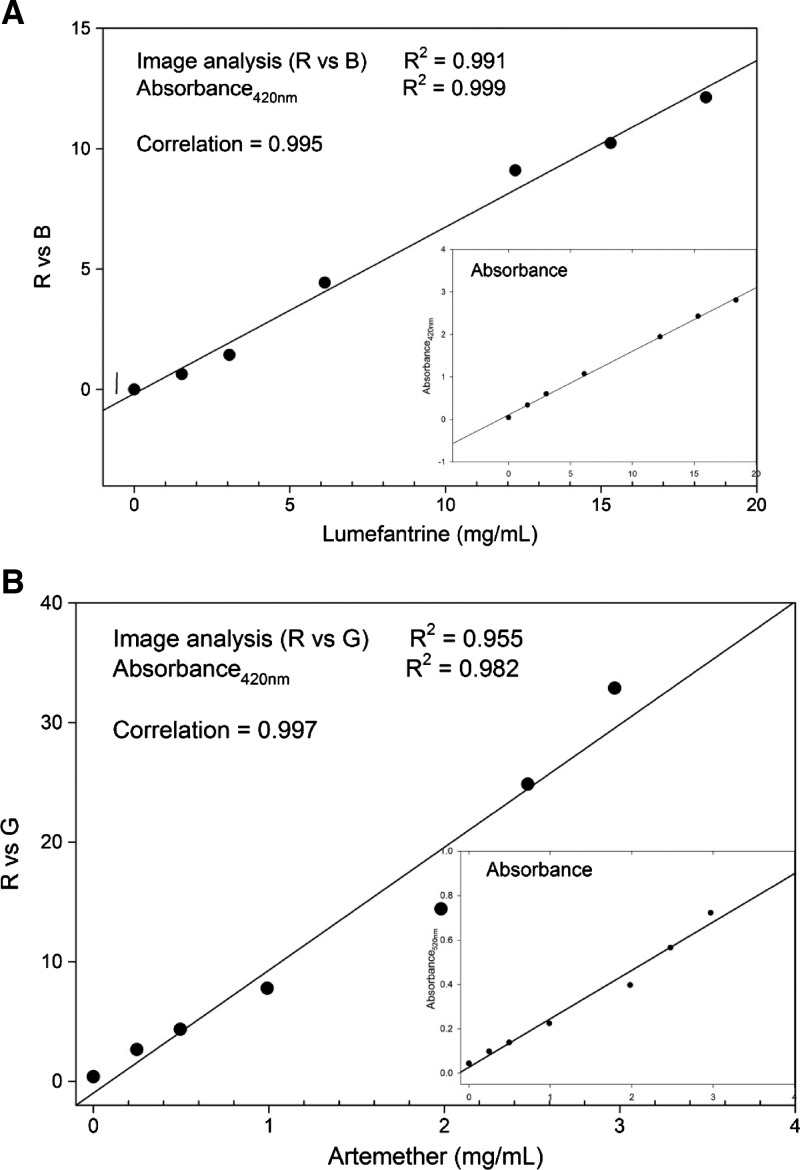
Comparison of the linearities and correlations of the colorimetric test for lumefantrine (**A**) and artemether (**B**) when analyzed by absorbance and image analysis techniques.

shows the linear relationship of the LF concentrations relative to absorbance (*R*^2^= 0.999) and R versus B (*R*^2^ = 0.991) of the acetic acid extract from an authentic pharmaceutical preparation. Good correlation (*R* = 0.995) between absorbance and R versus B pixel image analysis for LF was observed. Linearity of AM relative to concentration, and the correlation between absorbance and image analysis (R versus G) are shown in Figure 1B. Although the correlation between absorbance and R versus G was good (*R* = 0.997) for AM, there was more variability in the colored product (*R* = 0.955 for R versus G, *R* = 0.982 for absorbance) from the reaction of artemether with sulfuric/acetic acid. The specificity of the colorimetric method in terms of the observed color produced for common excipients and other commonly used drugs are listed in Table 3. Of the compounds tested, primaquine and tetracycline were soluble enough in acetic acid to produce a yellow/orange color while the slight solubility of amodiaquine resulted in a weak yellow color. On treatment with sulfuric acid, the color of the compounds remained; although less intense for primaquine. Artemisinin and its derivatives all produced a yellow/orange/red-colored product, while erythromycin turned black. The colorimetric assay was applied to a set of known counterfeits and compared with an authentic reference tablet (Figure 2A

**Figure 2.**
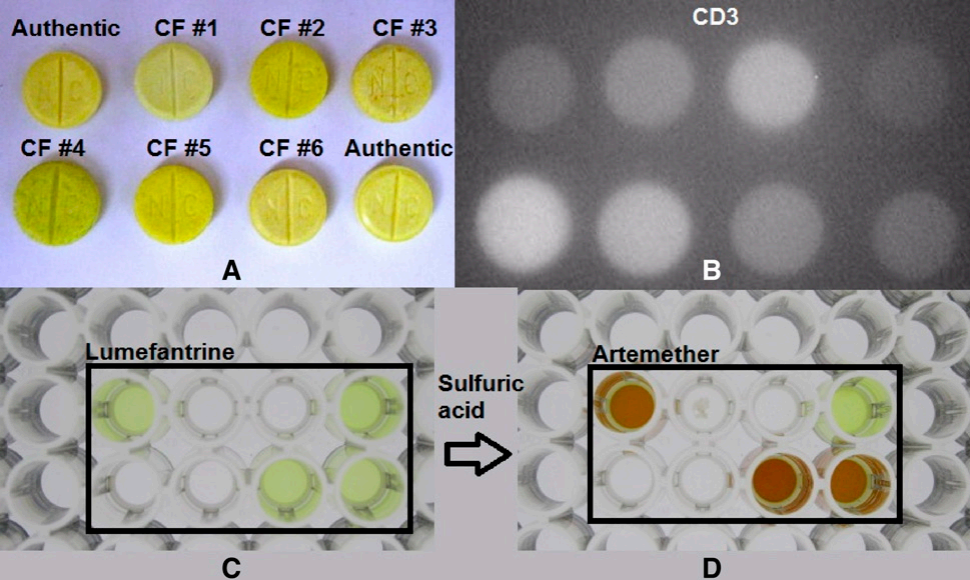
(**A**) Photograph of falsified (counterfeit [CF] #) and authentic tablets, (**B**) CD-3 visual fluorescence, (**C**) tablet material dissolved in acetic acid, and (**D**) subsequent colorimetric reaction of artemether with sulfuric acid.

). The acetic acid extracts and subsequent color reaction with sulfuric acid for this set of tablets are shown in Figure 2C and D, respectively. Although all the tablets appeared yellow, the yellow product was not soluble in acetic acid for samples CF #1, CF #2, CF #4, and CF #5. Sample CF #3 showed a yellow product, but was not LF as confirmed by HPLC and Raman spectroscopy, while the yellow product in sample CF #6 was confirmed to be LF at the proper concentration for an authentic product. Image analysis was performed on colors (R versus B for LF) and (R versus G for AM) from the samples in Figure 2C and D. Since the image analysis results were shown to be linear relative to concentration, the %API was calculated as a percent of the authentic reference tablet (Figure 3

**Figure 3.**
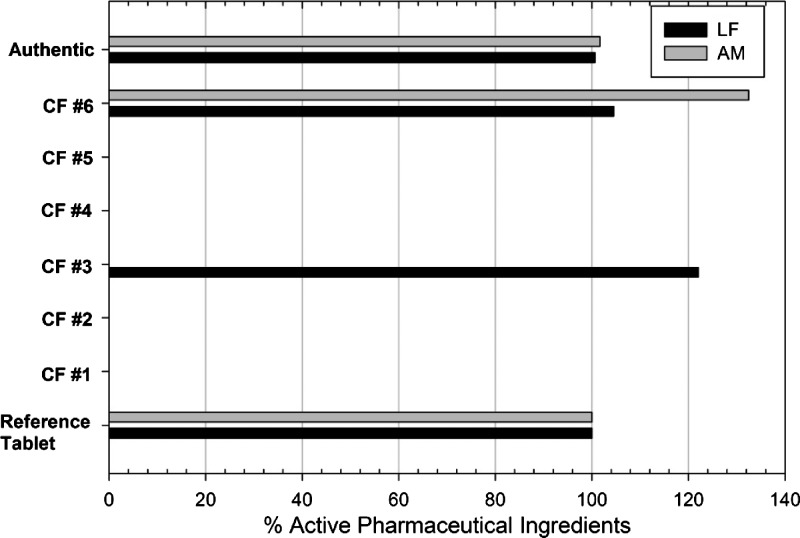
Colorimetric test intensities using image analysis techniques. Values determined by direct comparison with the authentic reference tablet.

). Since no colors were apparent for either LF or AM for CF #1, CF #2, CF #4, and CF #5, these samples were considered to be falsified products based on the absence of both active ingredients via the colorimetric assay. Sample CF #3 showed a yellow product equivalent to 121% LF and no artemisinin derivative. The colorimetric test indicated the absence of one of the active ingredients, and therefore this sample would be designated a counterfeited product. Analysis of CF #3 by HPLC and Raman spectrometry showed no detectable levels of LF. In this case, the yellow product in CF #3 is extractable in acetic acid and mimics the presence of LF. The colorimetric test showed CF #6 to contain both LF (111%) and AM (135%). HPLC analysis revealed this sample to contain appropriate amounts of both of the active ingredients, 108% LF and 112% AM. It is suggested that samples showing a positive colorimetric test should subsequently be analyzed by standard HPLC methods to determine accurate %APIs.

### CD-3 analysis.

Figure 2B is a digital photograph of the sample tablets taken with the CD-3 and shows the comparative intensities of the tablet fluorescence. Samples CF #1, CF #2, CF #4, CF #5, and CF #6 were noticeably brighter than the authentic tablets, while CF #3 was visibly darker. It should be noted that sample CF #6, which contained the correct amounts of active ingredients, was substantially brighter; indicating a suspected counterfeit. A CD-3 scan of the packaging revealed inconsistencies of various inks relative to the authentic reference package (image not shown for security purposes). A diode array UV spectrum of the chromatographic peak for artemether revealed contamination or possible degradation. It has been observed in our laboratory that artesunate tablets subjected to elevated temperatures tended to fluoresce more than samples kept under normal conditions. Therefore, it is suspected that sample CF #6 may have been an expired/degraded product, which may have been repackaged to resemble a legitimate product with an altered expiration date.

### CoDI analysis.

**Figure 4.**
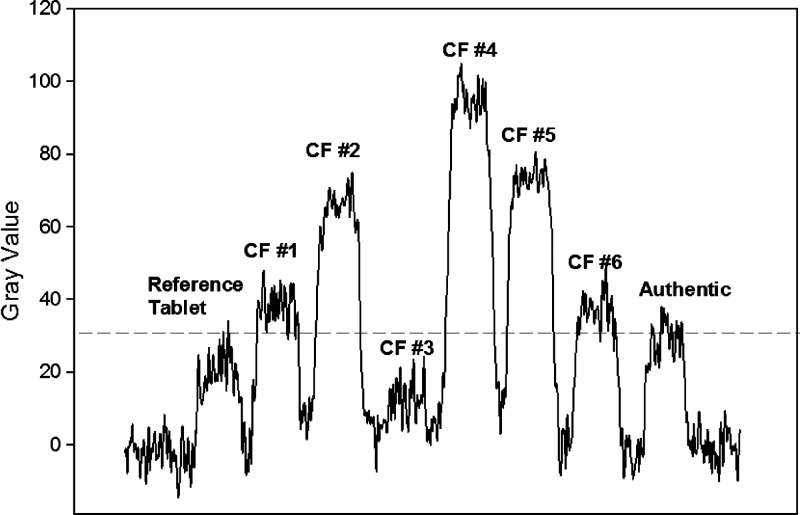
Counterfeit drug indicator (CoDI) box-and-whisker plot of “*W*” values [*W* = (*I* – *I*_filter_)/(*I*_filter_/*I*)] from 12 authentic Coartems and 6 counterfeited ones and their comparison with *W* values for other commonly used antimalarials. The *W* values for the counterfeits were well outside the range found for the authentics while all the *W* values of the other drugs (*N* = 5 for each brand/manufacturing) were significantly different (*P* < 0.002, Mann–Whitney test).

Figure 4 shows box-and-whisker plots for each group of drugs analyzed by the CoDI. The median value of “*W*” for the authentic Coartems is 1.88 (range: 1.30–3.25, *N* = 12). All counterfeit Coartems (CF #) were outside the range; including CF #6 (*W* = 0.30). All the groups of drugs were significantly different (*P* < 0.002, Mann–Whitney test) from the authentic Coartem group indicating this technique to be very specific to Coartem.

## Discussion and Conclusions

The main concerns for accurate field testing of antimalarials in resource-poor countries are the affordability and the regional availability of associated reagents and equipment and human capacity. The colorimetric test described in this article assesses the inherent yellow coloration of lumefantrine dissolved in acetic acid without the use of reactants, followed by the addition of sulfuric acid that reacts with artemether forming a red/orange reaction product. A search for African suppliers of chemicals through Alibaba.com, a leading platform for global wholesale trade, revealed 12 suppliers for concentrated sulfuric acid and 23 suppliers for glacial acetic acid. Therefore, these chemicals are assumed to be much more easily procured in Africa relative to the reactants Fast Red TR salt, dinitrophenylhydrazine, and Fast Blue RR used in other colorimetric methods for the artemisinins. A search for these reactants through Alibaba.com revealed no African suppliers. The acids used in the colorimetric test are caustic, therefore care should be used when performing the assay and personal protective equipment such as gloves and safety glasses are essential. The colorimetric reaction is based on the Zak reaction, which was initially applied to cholesterol analysis using acetic acid and sulfuric acid in the presence of Fe^3+^ as an oxidizing agent.^27^ Artemisinin and its derivatives are sesquiterpene lactone compounds possessing a reactive endoperoxide group. Therefore, this compound can act as its own oxidizing agent in the absence of Fe^3+^, making this reaction highly specific for the artemisinins (Table 3). It is suspected the colored reaction product is a conjugated polyene derived from oxidation and dehydration of the sesquiterpene lactone. Optimum intensity of the red/orange product occurs in 30–60 minutes and progressively turns to a dark brown at ambient temperatures. The color intensity is linearly proportional to the concentration, allowing direct proportional comparisons with an authentic reference standard tablet. The color intensity can be measured by image analysis of red, green, and blue pixels from a digital photograph or directly using smartphone image analysis applications. Image analysis techniques have been successfully used in colorimetric assays for the determination of deltamethrin levels on insecticide-impregnated mosquito nets.^28^,^29^ To minimize transportation and usage of the reagents as well as increase throughput, it is suggested the tablets be initially scanned using nondestructive techniques that do not require sample preparation (Tier II).

**Figure 5.**
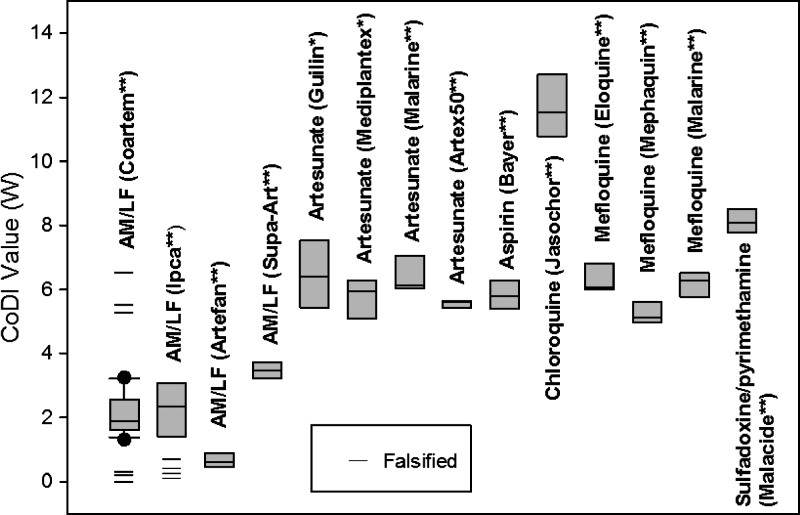
CD-3 fluorescent intensities as measured from the photograph (Figure 2B) using image analysis techniques. The level of intensity for authentic tablets including the reference is indicated by the dotted line.

The CD-3 has been fully evaluated in the field on artesunate samples collected in SE Asia.^25^ A total of 203 tablets were analyzed and compared with authentic tablets, resulting in a specificity, sensitivity, and positive predictive values of 100%, 98.4%, and 100%, respectively. All observations were made by visual interpretation with interobserver (*N* = 3) agreement of 100%. In this study, the CD-3 was used to evaluate a collection of confirmed falsified Coartem tablets relative to an authentic reference sample. Confirmation of falsified samples was established by Raman scans, HPLC, or inconsistencies of the packaging print relative to an authentic package. Although, the CD-3 has multiple LEDs that emit light of various wavelengths with both a visible and IR modes of detection, we used the UV LED as the light source in IR detection mode for the Coartem analysis (Figure 2B). The counterfeits were bracketed in the top left and bottom right corners by authentic tablets, which were stored under nominal conditions (i.e., in the blister pack at room temperature) and used within the expiry date. Visual interpretation clearly reveals differences in brightness relative to the authentic tablets. To allow for a more objective interpretation, we also incorporated image analysis techniques to compare the fluorescence intensity of the samples from a digital photograph (Figure 5). Image analysis software has been incorporated into a newer version of the CD-3, termed the CD-4. Note that tablet sample CF #6 is slightly more fluorescent relative to the authentics, although it has the correct amounts of active ingredients, as determined by HPLC analysis. Diode array analysis of the spectral purity associated with the artemether chromatographic peak showed a high level of impurities, suggesting a degraded product. Since the CD-3 scan of the packaging suggests a counterfeited product, it is assumed that expired/degraded Coartem tablets were repackaged with another expiration date. We observed that the fluorescence of artesunate tablets stored at higher temperatures (40°C) increased relative to those stored at ambient temperatures (25°C). Therefore, tablet fluorescence intensities may be affected by degradation products. Thus, the CD-3 may be useful in determining if medications have been stored properly. Authentic tablets typically produce consistent fluorescence intensities and any deviation would be considered as a suspected counterfeit. However, a counterfeited product can contain a variety of ingredients; therefore, a sample with similar intensity to the reference tablet does not necessarily indicate an authentic sample.

**Figure 6.**
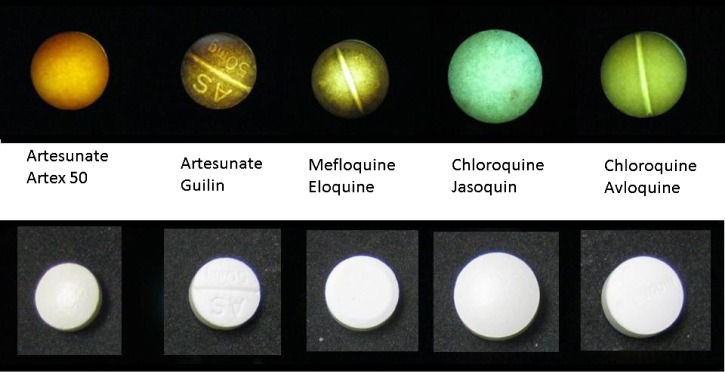
Colors produced by various antimalarials using the counterfeit drug indicator (CoDI), demonstrating its fluorescent properties when a near-ultraviolet (near-UV) laser is placed behind the sample tablet.

The CoDI is a novel device that measures the amount of laser light being transmitted through a tablet. Depending on the composition of the tablet, the use of a near-UV laser may result in a color change of the transmitted light. The change in the emitted wavelength (fluorescence) is characteristic of particular brands of tablets and can be used to distinguish these drugs from counterfeits or other brands. Figure 6 shows the various colors and intensities emitted from commonly used antimalarial drugs using the 405-nm laser placed behind the tablet. Under normal light, all these tablets appear white. Digital photographs of the emitted colors can be analyzed by image analysis software to distinguish between brands or identify counterfeits. Smartphone image analysis apps can provide rapid real-time color comparisons of samples.

An objective of this article is to illustrate the use of multiple, simple, and low-cost techniques to accurately identify a counterfeit drug. Here, we use the popular and effective antimalarial drug Coartem as an example. We recommend a three-tiered system: 1) physical properties of a tablet (e.g., weights and dimensions), 2) photometric techniques for rapid scanning (e.g., CD-3 and CoDI), and 3) colorimetric techniques for identification and quantification of active ingredients. Chemicals used for the colorimetric tests (Tier III) are conserved, since it was used only for the samples failing the photometric scans (Tier II). Table 4 gives a summary of the test results for six counterfeited Coartem tablets. Tier I tablet measurements show CF #2 and CF #6 to be within acceptable limits, while Tier III colorimetric results show CF #6 to pass quantitative limits for the active ingredients. Tier II qualitative photometric analysis was able to distinguish all the samples as suspicious, that is, counterfeited or degraded. It is recommended that liquid chromatography–mass spectrometry (LC-MS) techniques be used for confirmation.^30^

## Figures and Tables

**Table 1:** Comparison of tests in terms of tier category

Test type	Type of assessment	Level of training*	Facility	Equipment	Cost (USD)	Requires sample preparation	Active ingredient identification
Physical (Tier I)							
Dimensions	Quantitative	Inspector*	Field	Micrometer or caliper (0.1 mm)	< 100	No	No
Weight	Quantitative	Inspector*	Field	Portable balance (0.001 g), batteries	< 100	No	No
Visual color	Qualitative	Inspector*	Field	None	0	No	No
Photometric (Tier II)							
Raman (portable)	Qualitative	Inspector†	Field	Device, batteries	> 10,000	No	No
NIR (portable)	Qualitative	Inspector†	Field	Device, batteries	> 10,000	No	No
CD-3	Qualitative	Inspector†	Field	Device, batteries	∼1,000	No	No
CoDI	Qualitative	Inspector†	Field	Device, batteries	∼100	No	No
Chemical (Tier III)							
TLC	Qualitative	Laboratory technician‡	Basic laboratory	TLC plates, labware, developing chamber, sprayer, chemicals	< 100	Yes	Yes
	Quantitative						
	Semi-quantitative						
Colorimetry (see Table 2)	Qualitative	Laboratory technician‡	Basic laboratory	Labware, chemicals	< 100	Yes	Yes
	Quantitative						
	Semi-quantitative						

CD-3 = counterfeit detection device version 3; CoDI = counterfeit drug indicator; NIR = near-infrared; TLC = thin-layer chromatography; USD = U.S. dollars.

* Little or none.

† Moderate.

‡ High.

**Table 2:** Comparison of tablet measurements

Falsified tablets	Weight (0.228–0.252 g)*	Diameter (9.1–9.2 mm)†	Thickness (3.0–3.4 mm)*	Pass/Fail‡
CF #1	***306***	9.2	3.4	Fail
CF #2	241	9.2	3.2	Pass
CF #3	343	9.2	***3.7***	Fail
CF #4	***304***	***10***	***3.7***	Fail
CF #5	***260***	***9.3***	***3.5***	Fail
CF #6	243	9.2	3.2	Pass

CF = counterfeit.

Bold/italic figures were used to emphasize values that were out-of-range for the Coartem specifications.

* Coartem tablet specifications per Novartis personal communication.

† Range as determined from 12 authentic tablets.

‡ Pass = tablet measurements within all three ranges; fail = tablet measurement outside of at least one range.

**Table 3:** Specificity of the colorimetric test to common excipients and other antimicrobials

Compound	Acetic acid extract	Sulfuric acid treatment
Excipients		
Microcrystalline cellulose	Colorless	−
Croscarmellose	Colorless	Colorless
Fumarate	Colorless	Colorless
Talc	Colorless	Colorless
Lactose	Colorless	Colorless
Starch	Colorless	Colorless
SiO_2_	Colorless	Colorless
Mg stearate	Colorless	White precipitant
Dihydroartemisinin	Colorless	Orange/red
Artesunate	Colorless	Red
Artemether	Colorless	Orange/red
Artemisinin	Colorless	Yellow/orange
Sulfamethoxazole	Colorless	Colorless
Acetaminophen	Colorless	Colorless
Aspirin	Colorless	Colorless
Pyrimethamine	Colorless	Colorless
Trimethoprim	Colorless	Colorless
Chloroquine	Colorless	Colorless
Chloramphenicol	Colorless	Colorless
Erythromycin	Colorless	Black
Ciprofloxacin	Colorless	Colorless
Primaquine	Yellow/orange	Weak yellow
Ampicillin	Colorless	Colorless
Sulfadoxine	Colorless	Colorless
Tetracycline	Yellow/orange	Orange
Mefloquine	Colorless	Colorless
Amoxicillin	Colorless	Colorless
Piperaquine	Colorless	Colorless
Amodiaquine	Weak yellow	Weak yellow
Lumefantrine	Yellow	Yellow

**Table 4:** Summary of test results

Sample	Tier I (tablet measurement)	Tier II (CD-3)	Tier II (CoDI)	Tier III (colorimetric)
CF #1	Fail	Fail	Fail	Fail
CF #2	Pass	Fail	Fail	Fail
CF #3	Fail	Fail	Fail	Fail
CF #4	Fail	Fail	Fail	Fail
CF #5	Fail	Fail	Fail	Fail
CF #6	Pass	Fail	Fail	Pass

CD-3 = counterfeit detection device version 3; CoDI = counterfeit drug indicator; CF = counterfeit.
